# What Eugenics Does Not Claim to Do

**Published:** 1912-09

**Authors:** 


### What Eugenics Does Not Claim To Do.

Eugenics does not mean to develop a uniform single type of man, or to breed men as we do cattle. It wisely recognizes the broad distinction between the human and the animal kingdoms and the impracticability of applying the same rules to both. Society needs the different types of men, and it will be the aim of this new science to maintain the various types in their greatest purity.

Likewise, eugenics does not aim to deliberately breed characters—like genius, patriotism, agility and the like; such inherent traits will be improved by the better environments offered.

Eugenics will not deny a man his freedom in the choice of a mate within the confines of what society would prescribe as essential to its welfare. It does not infringe on his personal rights, for these are restricted by due consideration of others. Nor are compulsory marriages, without love, contemplated, for the eugenic idea is based on sexual selection under improved environments. Love is inherent in the breast and can not be repressed, though marriage has always been restricted since the existence of society.

Eugenics does not oppose religion by conforming to natural impulses; it does not mean to suggest a pessimistic view of life by emphasizing the prevalence of disease and crime; nor does it deny the poor the right of parenthood because of their social condition, but insists on the best of each class to become the fathers and mothers of the next generation.

